# Psychosocial treatments for nightmares in adults and children: a systematic review

**DOI:** 10.1186/s12888-023-04703-1

**Published:** 2023-04-21

**Authors:** Peter Gill, Emily Fraser, Thong Thai Diep Tran, Gabriel De Sena Collier, Andrew Jago, Joe Losinno, Michael Ganci

**Affiliations:** 1 Institute for Health and Sport, Victoria University Australia, Footscray Park, Ballarat Rd, Melbourne, VIC Australia; 2 Turner Institute for Brain and Mental Health, Monash University, Melbourne, Australia

**Keywords:** Nightmares, Psychosocial treatments, PTSD, CBT

## Abstract

**Background:**

As nightmares may be a risk factor for, or symptom of, multiple psychological disorders, some researchers suggest that nightmares should be screened, diagnosed, and treated. Treatments for nightmares include trauma-focused Cognitive Behavioural Therapy and Image Rehearsal Therapy, and pharmacological interventions such as prazosin and nitrazepam. As recent research has put into question our current understanding of treatment efficacy, there is a need to systematically review findings related to the effectiveness of nightmare treatments to inform best practice. The current review assessed the efficacy of psychosocial treatments of nightmare in all cohorts.

**Methods:**

A systematic search of four databases for peer reviewed journal articles from 2000 onwards produced 69 (35 RCTs, 34 non-RCTs) eligible articles that underwent narrative synthesis.

**Results:**

The results provide strong evidence for exposure and image rehearsal treatments for the reduction of nightmare frequency, severity, and distress, in civilian, military, idiopathic, and posttraumatic stress disorder (PTSD) cohorts. There is emerging evidence that self-guided and brief treatment modalities offer efficient and effective treatment options. There is an urgent need for clinical trials of treatment effectiveness in children.

**Conclusions:**

The results suggest that treatments for nightmares are most effective when they facilitate a sense of control or mastery by directly targeting the nightmare content and/or the client’s emotional responses to the nightmare content.

**Trial registration:**

A review protocol was registered with PROSPERO (CRD42020204861).

## Background

According to the DSM-5, nightmares are an intrusive dream and may be idiopathic or associated with disorders such as nightmare disorder, PTSD, substance abuse, and schizophrenia [1]. As nightmares may also be a risk factor for PTSD, and can be present after the successful treatment of PTSD, some researchers suggest that nightmares should be screened, diagnosed, and treated [2]. Furthermore, the International Classification of Sleep Disorders (ICSD) recognize and describe nightmare disorder as a REM parasomnia, affecting more than five percent of the US population [3]. Nightmare disorder is related to repeated (at least once per week), well remembered nightmares that result in rapid awakening, and mood, sleep, and behavioural problems more generally [4]. PTSD related nightmares can be considered a distinct subset of nightmare disorder [5]. Researchers believe there may be a genetic component to nightmares and a strong association with neuroticism and trauma [6]. Since 2000, research has increasingly considered nightmares as a complex phenomenon with multiple presentations and a potential stand alone disorder, rather than just a symptom of sleep disorders as previously thought.

Currently, nightmare disorders are under diagnosed, and may be prevalent in as many as 5% of the population. Nightmares have also been shown to increase the risk of suicide behaviours in depressed patients [7], and are associated with increased interpersonal violence [8]. Frequent nightmares may relate to a five-fold increase in the likelihood of having a psychiatric illness [3, 9]. Nightmares are reported more commonly by women, but no sex differences occur in children or in older adults [10]. Nightmares have also been reported across multiple geographic and cultural contexts. Due to the high prevalence of nightmares in all populations and their links to psychopathology it is important that we develop and evaluate evidence-based treatments.

Studies on nightmares and nightmare treatment in children are currently limited, especially younger children, with prevalence rates estimated to be similar to adults (approximately 5%) [11]. Nightmares in children are associated with a wide range of sleep related, emotional, developmental, and behavioral problems [12]. In a sample of treatment seeking war-exposed youths, nightmares were associated with significant suffering, with the most common nightmare themes being fear (77%), grief (20%), and hopelessness (19%)  [13].

## Psychosocial treatments

The results of meta-analytic reviews suggests that psychosocial treatments can reduce nightmare frequency and intensity with medium effect sizes commonly reported [14, 15]. Systematic reviews support the efficacy of trauma focused CBT, especially image rehearsal therapy (IRT), for nightmares, however there are limited high quality clinical trials [16–18]. The American Academy of Sleep Medicine (AASM) recommend image rehearsal therapy (IRT) for PTSD related nightmares and nightmare disorders, and also list a number of “may be used” treatments such as cognitive behavioral therapy (CBT), eye movement desensitization and reprocessing (EMDR), and exposure, relaxation, and rescripting therapy (ERRT) [19]. They also stress the importance of tailoring treatment to the specific client and context. The British Association for Psychopharmacology review of treatments recommends psychosocial interventions for nightmares that include exposure, writing down dreams, guided imagery, pleasant images, and changing the ending [20]. There are however some inconsistencies and unknowns in the current guidelines. For example, CBT is considered a first line treatment by the British Association for Psychopharmacology, but a second line treatment by AASM. There is also a lack of evidence for psychosocial treatments for severe nightmares and severe nightmare disorders. Most of the current trials are mild to moderate cases.

Rousseau and Belleville [21] systematically reviewed the mechanisms by which nightmares are treated psychosocially. They concluded that an increased sense of mastery was the most commonly cited explanation for therapeutic benefits. Reductions in arousal, fear and avoidance, improved sleep, and modification of beliefs were also cited as mechanisms of action for psychosocial interventions for nightmares.

Consistently, IRT is listed as a first line treatment. IRT involves the client writing down the dream or drawing it in the case of children. The client is then encouraged to imagine themselves acting differently. This often involves imagining an action or series of actions that replace non-action. This encourages a re-imagining where action is taken rather than flight. The client can write or draw these alterations, and are encouraged to repeat this process consistently at home  [6].

### Pharmacological treatments

There is also extensive research on drug treatments for nightmares. According to Morgenthaler et al., [19] pharmacologic treatment may be slightly more effective than psychosocial treatments. They also suggest that there is conjecture over the lasting effects of these drugs once withdrawn, and that we are still unclear about the underlying pathophysiology of nightmares. There is also a need to better understand interaction effects between anti-depressants and nightmare specific drugs such as prazosin. The AASM currently suggest that the following drugs may be beneficial for treating nightmares; the atypical antipsychotics olanzapine, risperidone, and aripiprazole; clonidine; cyproheptadine; fluvoxamine; gabapentin; nabilone; phenelzine; prazosin; topiramate; trazodone; triazolam; nitazepam; and tricyclic antidepressants. One of the most widely studied, reviewed and utilized treatment drugs for nightmares is the alpha-1 adrenergic blocker prazosin [22]. The British Association for Psychopharmacology review of treatments stated that there was good evidence for the use of prazosin for reducing nightmares in adult and pediatric populations [20]. However, while still supporting its use, the AASM downgraded the effectiveness of prazosin for nightmares in 2018.

Suraev et al [23] and Betthauser et al [24] systematically reviewed cannabinoid therapies for managing sleep disorders and despite some promising preliminary evidence, both studies suggested that there is currently an absence of high quality clinical trials to support its use. Cowling and MacDougall [25] reported that the synthetic cannabinoid nabilone could reduce PTSD related nightmares. Dagan and Yager [26] argued that while medical cannabis could reduce nightmares, it may have negative effects on other PTSD symptoms such as dissociation and reckless behaviours. There is also some evidence for the use of anti-depressants in treating nightmares. A systematic review of the links between dreaming and anti-depressants found differing effects depending on the type of anti-depressant taken, including withdrawal effects, and concluded that more research is needed to draw conclusions [27]. In addition, side effects of some anti-depressents include an increase in nightmares. In summary, there is limited evidence supporting the use of cannabis or anti-depressants for nightmares. However, as an emerging area of investigation, an up to date review of research findings is needed.

In summary, treatment for nightmares and nightmare disorder include psychotherapeutic treatments such as image rehearsal therapy, pharmacological interventions such as prazosin and nitrazepam that affect the neurotransmitters, and atypical antipsychotics such as olanzapine. In recent times there have been numerous studies that have evaluated the effectiveness of treatments for nightmares, resulting in some changes to understanding of best practice. For example, some psychosocial treatments recently trialed include eye movement desensitization and reprocessing, and sleep dynamic therapy. In 2018, the AASM released a position paper (rather than a clinical practice guide) due to the limited number of high quality studies providing direct evidence of treatments for nightmares [19]. In addition, many studies in this area have evaluated treatments for sleep related outcomes in general, rather than treatments for nightmares specifically. There is also a lack of a universal outcome measure for nightmares which makes comparing study results difficult. As such there is a need to systematically review findings related to the effectiveness of treatment of nightmares to inform best practice. This current study reviewed psychosocial treatments for nightmares and adds to the review literature by including non-RCTs, studies on children, and a focus on newer (for nightmares) psychosocial treatments. We aimed to answer the following questions:

What is the effectiveness of psychosocial treatments for nightmares in adults and children?

What are the new promising psychosocial nightmare treatments for adults and/ or children requiring further investigation?

## Method

This report followed the systematic review reporting guidelines suggested by Moher et al. [28] A review protocol was registered with PROSPERO (CRD42020204861). This paper reported the psychosocial findings from this protocol.

### Searching the literature

An initial search was conducted to find current meta-analytic, systematic review, and review articles using SCOPUS (2000-Present), PsychINFO (2000-Present), and MEDLINE (2000- Present) databases and the search terms “nightmare AND review OR analysis”. This informed the focus of the current review and the information presented in the introduction section of this report. A search was performed to collect relevant studies for the systematic review. The databases SCOPUS, PsychINFO, CINAHL, and MEDLINE were searched using the search terms “nightmare AND therapy OR treatment OR intervention”. Relevant database subject heading search terms were also included. In addition, the Cochrane data base was searched for trials (*n* = 582) and reviews (*n* = 65) using the search term “nightmare”.

Reference lists of more recent studies were screened for studies not picked up by the search. The final search was performed on August 30, 2020.

The following inclusion criteria was used to screen studies:

1. Available in English or English translation.
2. Published in 2000 or later. Prior to 2000, research more commonly considered nightmares as a symptom of sleep disorders more broadly rather than a stand-alone disorder.
3. Reported findings related to the effectiveness of psychosocial or pharmacological treatments.
4. Included any nightmare symptoms reported as an outcome variable, either as a specific focus of the study such as in nightmare disorder, or as part of a broader outcome evaluation such as sleep disturbance or PTSD.
5. A peer reviewed scientific journal article.

Excluded from the review were review articles, theoretical or commentary articles, books, audio documents, posters, symposiums, and classification manuals. For this manuscript, only studies evaluating psychosocial treatments were presented.

### Study selection and data extraction

The first author reviewed all eligible studies. Studies were screened at two stages, title and abstract, and full text. Eligibility assessment was performed independently in a blind standardized manner by two reviewers for title and abstract, and one reviewer for full text using the software COVIDENCE. Overall agreement rates were 84.5%, for title and abstract screening with discrepancies solved through discussion in regular meetings between the two reviewers.

Data were extracted by two reviewers, and included the following:

1. Study, authors, date, and country
2. Sample characteristics (age, sex, military or civilian, nightmare severity/ presentation)
3. Study design/ protocol (experimental, RCT, trial, case series, pilot)
4. Outcome measures; nightmare frequency (number of nightmares, number of nights with nightmares), nightmare severity (intensity, distress caused)
5. Treatment type/ format/ length/ duration
6. Attrition rate/percentage
7. Outcomes (effects within and between groups, including follow up)
8. Study limitations

### Risk of bias within studies

A process for assessing bias within RCT studies was formulated according to Cochrane recommendations [29] and using the COVIDENCE software template. For RCTs an assessment for each study was made and reported as either low risk, high risk or unclear risk;

1. Selection, allocation, group differences at baseline
2. Blinding of participants, personnel, and outcome assessment, non-planned treatment differences
3. Differences in how group outcomes were assessed, non-validated measures of nightmares
4. Incomplete outcome data, Attrition differences between groups
5. Selective Outcome Reporting

For non-RCTs, several tools were considered including the Risk of Bias in Non-Randomized Studies—of Interventions (ROBINS-I) [30], and the Joanna Briggs Institute (JBI) critical appraisal checklist for case reports [31]. However, due to the variability of study designs included, a selection of relevant criteria were assessed for each study and reported as either low risk, high risk, or unclear risk. These assessments were made based on a comparison to the other non-RCTs in this collection rather than in comparison to the stringent RCT expectations.

1. Participant selection – minimum frequency of nightmares stated or met (once per week)
2. Confounding variables – concurrent psychological or pharmacological treatment
3. Blinding of outcome assessors
4. Selective Outcome Reporting
5. Data Sampling

The percentage of published articles with significant findings were also considered along with collective estimates of conflicts of interest and funding sources.

### Data synthesis

Data synthesis was performed narratively. Due to the diverse outcome measures, treatments, and samples evident in the literature on nightmares, a focused narrative approach allowed for the best synthesis of the data. Studies were grouped into main categories in relation to the type of treatment, including group or individual, type of psychosocial treatment, and also by age, adults and children. Further sub-categories were devised for grouping treatment types; CBT based, Exposure based, Image rehearsal/rescripting based. Once all articles were grouped into categories, the collective efficacy was assessed. This process was completed in discussions between two of the researchers and resulted in therapies being classified at low, medium, or high on quantity of evidence, quality of evidence, and support for use. This process was informed by the effects reported and the risk of bias. A conclusion was generated for all groups and sub-groups and then reported.

### Changes to PROSPERO

The primary change to this study was in study selection. Full text reviewing was conducted by one reviewer rather than two reviewers. This occurred due to the withdrawal of one of the reviewers from the research. It was decided to proceed with the primary reviewer alone rather than include a new reviewer who was less familiar with the studies. Due to the large number of studies included, the decision was made to report the study in two separate papers (psychosocial and pharmacological). We acknowledge that comparisons between the two broad treatment modalities are important, and will be discussed in a subsequent manuscript.

## Results

### Description of selected studies

**Fig. 1:**
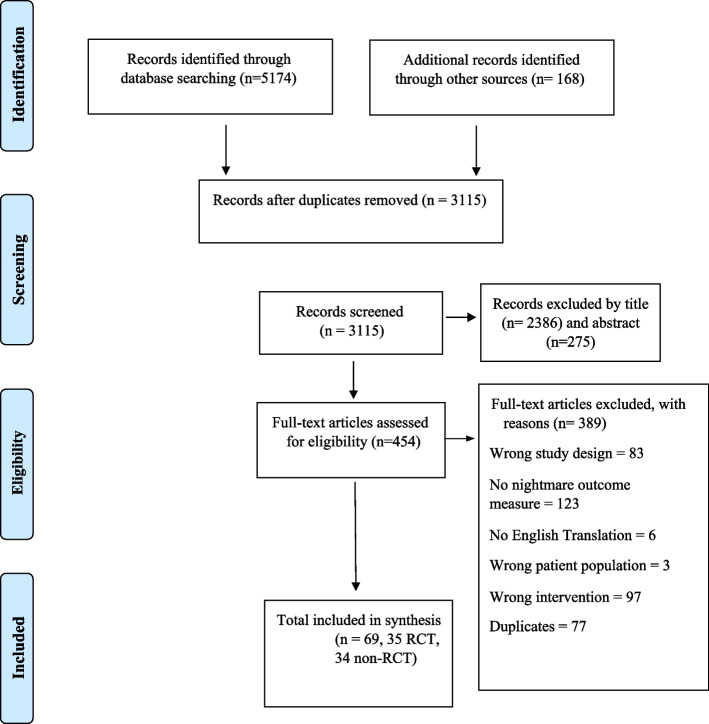
PRISMA Flowchart of Literature Search and Selection

The strategy for literature search and selection is outlined in Fig. 1. Title and abstract review resulted in 454 eligible studies. After excluding 389 studies for the reasons listed in Fig. 1, there remained 65 studies for the qualitative synthesis. The 35 RCT studies comprised a combined 3048 participants (F = 1940, M = 1108), with mean ages ranging from 10 to 59 years. The 34 non-RCT studies comprised a combined 500 participants (F = 170, M = 327, Transgender = 3) with ages ranging from 4 to 71 years. Only two RCTs and 7 non-RCTs included children and/or adolescents. Most RCT studies originated from either the USA (*N*=19) or the Netherlands (*N*=7), and 12 of the studies had military or military veteran samples. For the non-RCT studies, most studies originated from the USA (*N* = 27) and had civilians with PTSD (*N* = 11) or military or military veteran sample with PTSD (*N* = 13). Participants were most commonly suffering from PTSD (*N* = approximately 1880) or idiopathic (*N* = approximately 450) nightmares. Female participants were commonly victims of sexual assault, while males commonly suffered from war related trauma. The most common therapies evaluated were image rehearsal/rescripting-based therapies (*N*= 34), exposure-based therapies (*N*= 8), mixed exposure and image rehearsal based therapies (*N*=11), and CBT based therapies (*N*=6). The interventions consisted of individual, group, and self- help/directed modalities, ranging from 1 to 20 sessions in total, with each session lasting between 60 and 180 minutes. Attrition rates for the 35 RCT studies ranged from zero to 46% from baseline to final measurement. For the non-RCT studies, attrition rates ranged from zero to 64.7%. Most studies included follow up measurement/s of between 4 weeks and 12 months. The nightmare related outcomes measured included frequency (total number, number of nights with), intensity, and related distress, measured by self-report or a standardized instrument. The majority of RCT studies assessed both within and between group effects using analysis of variance, whereas most of the non-RCTs did not use a comparison group, assessing within-subjects effects only.

### RCTs

One study (see Table 1) assessed the efficacy of treatment for children [32] and found that a self-help form of IRT significantly reduced nightmare frequency compared to a passive control (sustained at 9 month follow up). Another study assessed IRT with an adolescent (13 years to 18 years) female only cohort and found significant reductions in nights and nightmares per month compared to no significant changes in the control group [33].

**Table 1:** Summary of RCTs examining psychosocial treatments of nightmares

Author	Country	Participants/ Sex/ *N* = female	Mean Age	Population	Intervention	Control	Sessions	Mode	Outcome measures	% Attrition	Outcomes
Belleville et al [34]	Can	42 (37 F)	30	Civ PTSD	CBT + IRT	Active (CBT)	5 × 60 min	Ind	NDQ: DI, NNN	7	CBT and CBT + IRT sig decreased NM symptoms (DI but not NNN). Adding IRT early sig better than CBT alone. No F/U
Casement et al [35]	USA	45 (0 F)		Civ PTSD	NET	Passive(waitlist)	6 weeks	Ind	NN		NET sig reduced NN (vs control) post-treatment. Maintained at 3 m F/U
Cook et al [36]	USA	124 (0 F)	59	Mil PTSD	IRT	Active (sleep & NM Man)	6 × 90 min	Group	NFQ: NN, NNN NES: DI	10.5	No sig effects including 6 m F/U
Davis et al [37]	USA	47 (35 F)	47	Civ PTSD	ERRT	Passive (waitlist)	3 × 120 min	Ind Group	TRNS: NN, NNN, IN	25.5	Sig improvements found for NN, NNN & IN at 1w post-test and at 6 m F/U
Davis & Wright [38]	USA	49 (40 F)	40	Civ PTSD	ERRT	Passive (waitlist)	3 × 120 min	Ind Group	TRNS: NN, NNN, IN	25.6	84% reported absence of NM in previous week at 6 m F/U. Most effective on IN
Forbes et al [39]	AUS	12 (0 F)	48	Mil PTSD	IRT	No control	6 × 90 min	Group	NN, NNN, IN	0	NN, NNN, IN improved sig (vs control) post treatment, and at 3 m & 12 m F/U
Germain et al [40]	USA	57 (12 F)	41	Mil PTSD	BSI (IRT & stimulus control/sleep restriction)	Active (Prazosin) or placebo	8 × 45 min	Ind	NNN	28	Sig reduction in NNN in both groups compared to control. CBT & Prazosin had an equivalent effect. No F/U
Gieselmann et al [41]	Germany	127 (109 F)	36	Civ Ideopath	IRT guided or IRT unguided	Active (frequency or narrative control)	6 sessions	Ind	NDQ: DI NN	28	Both guided & unguided better than controls for reducing NN & DI, except for NN for narrative control group. Effects held at F/U except narrative control group
Gray et al [42]	USA	74 (0 F)	49	Mil PTSD	RTM	Passive (waitlist)	3 × 120 min	Self-help	PSS-I: NN, DI	42	Sig reductions compared to control at post-test & 6w F/U
Gutner et al [43]	USA	171 (171 F)	32	Civ PTSD	CPT or PE	Passive (waitlist)	12 sessions	Group	NN, IN	29.2	Both CPT & PE significantly reduced NN & IN compared to waitlist, including 9 m & long-term F/U. No remission
Harb et al [44]	USA	108 (15 F)	37	Mil PTSD	IRT + CBT-I	Active (CBT-I)	6 × 60 min	Ind	NDQ: DI NFQ: NN NFQ: NN, NNN	28	Both groups showed 29% reduction in NN, NNN, with 22% remission. Combined therapy not better than CBT-I alone. No F/U
Holzinger et al [45]	Aus	40 (24 F)	35	Mil	Gestalt + LDT	Active (Geastalt)	9 × 90 min	Group	NN, NNN	20	Sig reduction of NM frequency was found in both groups after the 10w study & at F/U
Krakow et al [33]	USA	30 (30 F)	15.6	Civ PTSD	IRT	Passive (waitlist)	1 × 6 h day workshop	Group	NN, NNN	33	At 3 m, S-Rep, retrospectively assessed NNN sig decreased 57% with large effect size + NN sig decreased 71% with large effect size in the treatment group. No sig changes in the control group
Krakow et al [46]	USA	168 (168 F)	36	Civ PTSD	IRT	Passive	2 × 180 min + 1 × 60 min	Group	NFQ: NN, NNN NDQ: DI	40.5	Treatment sig reduced NN & NNN at post-test & 6 m F/U compared to control (moderate effect compared to small)
Kunze et al [47]	Neth	104 (80 F)	35	Civ Ideopath	IR IE	Passive (waitlist)	3 × 60 min	Ind	NN, NNN, DI	11.5	Compared to control, both groups sig reduced NN, NNN, & DI. Maintained at 6 m F/U. No diff between IR and IE. Effects mediated by increased mastery
Lancee et al [48]	Neth	70 (67 F)	30	Civ Ideopath	IRT	Passive (waitlist)	3 sessions	Guided self-help	SLEEP-50: DI, NN, NNN	17.1	Compared to controls IRT sig reduced NN, NNN, and DI, including 3 and 6 m F/U. Effects mediated by increased mastery
Lancee et al [49]	Neth	198 (159 F)	39	Civ	IRT or PE	None	6 weeks	Guided self-help	SLEEP-50: DI, NN, NNN	0	NN, NNN, DI had moderate effect sizes on both treatment conditions. No diff between conditions. Effects sustained at 42w F/U
Lancee et al [50]	Neth	278 (212 F)	36	Civ	IRT IRT + LDT + IRT	Passive (waitlist)	6 weeks	Guided self-help	SLEEP-50: DI, NN, NNN	45.7	Only IRT sig better than control. IRT better than IRT + and LDT + IRT. Consistent at 42w F/U, but high attrition
Lancee et al [51]	Neth	399 (307 F)	39	Civ	IRT or PE or Recording	Passive (waitlist)	6 weeks	Guided self-help	SLEEP-50: NN, NNN, DI	29.3	IRT & PE sig better than Recording in reducing DI, NN, and NNN. IRT best for NN, NNN, & PE best for reducing DI. Recording better than control. No F/U
Larsen et al [52]	USA	108 (108 F)	32	Civ PTSD	CPT or PE	None	-	Ind	NN, NNN, IN		Both CPT & PE sig reduced symptoms. No F/U. Guilt was the prevailing residual symptom
Margolies et al [53]	USA	40 (4 F)	38	Mil PTSD	CBT-I + IRT	Passive (waitlist)	4 × 60 min	Ind	NN, NNN, IN	25	Sig reduction in NN, NNN, IN compared to control. No F/U, high attrition
Pruiksma et al [54]	USA	70 (50 F)	43	Civ Ideopath	ERRT + Exposure and Rescripting	Active (ERRT w/o Exposure & Rescripiting)	3 × 90	Ind	NN, NNN, DI	-	Both groups showed medium to large effect size improvements in NN, NNN, DI. Conditions did not differ at any time point. Exposure and Rescripting did not add sig benefit. Benefits maintained at 6 m F/U
Pruiksma et al [55]	USA	40 (0 F)	33	Mil PTSD	ERRT-M	Active (MCC followed by EERT-M)	5 sessions or 5 weeks of control	Ind	NN, NNN, IN		Medium effect size reductions in NN, NNN, and IN. ERRT sig better than control at post-treatment & 1 m F/U
Rhudy et al [56]	USA	40 (29 F)	38	Civ PTSD	ERRT	Passive (Waitlist)	3 × 120 min (2 h per week)	Ind	Physical & emotional reactions	22.5	Treatment reduced physiological & subjective reactions to NM imagery compared to controls at post & 3 m F/U
Sheaves et al [57]	UK	24 (10 F)	41	Civ Psychosis	CBT + IRT	Active (IRT)	4 × 60 min	Ind	IN, NN, DI	0	NM specific CBT + IRT led to large improvements in NM compared to IRT alone. Gains maintained at 4w F/U compared to IRT alone
Spoormaker & van den Bout [58]	Neth	23 (17 F)	28	Civ PTSD	LDT 2 h (Individual) LDT 2 h (Group)	Passive (Waitlist)	1 × 120 min	Ind/Group	SLEEP-50: NN, IN	0	At 12w F/U, LDT groups sig reduced NN. Reductions also occurred in control
St-Onge et al [32]	Can	20 (9 F)	10	Civ Ideopath (Children)	IRT	Passive (Waitlist)	3 meetings	Self-Help	S-Rep, NDQ: NN, DI	0	IRT reduced NN compared to control including 9 m F/U. Pts had low numbers & IN at baseline
Swanson et al [59]	USA	10 (0 F)	59	Mil PTSD	CBT-I + ERRT	No control	10 × 90 min	Group	Diary—IN NN	20	Pts reported an average 50 % decrease in NN per week over 10w & large effect in reducing IN
Talbot et al [60]	USA	45 (31 F)	37	Civ PTSD	CBT-I	Passive (waitlist)	8 sessions (8 weeks)	Ind	IN, NN	26	CBT- I sig reduced NN & IN including 6 m F/U. Unclear evidence as to whether the effect was greater than control
Taylor et al [61]	USA	128 (19 F)	34	Mil PTSD	PE (Spaced)	Active PCT + PE (Massed)	10 sessions (2 or 8 weeks)	Ind	IN, NNN		Sig reduction in IN for the spaced group but not the massed group. Unclear comparisons to PCT
Thünker & Pietrowsky [62]	Germany	69 (47 F)	38	Civ Mixed	IRT	Passive)	8 × 50 min (10 weeks)	Ind	NN (Unknown Scale)	17 2	NN sig reduced over time including F/U, but effect was not greater than control
Ulmer et al [63]	USA	22 (7 F)	46	Mil PTSD	CBT + IRT	Passive Active	6 × 60 min (12 weeks)	Ind	S-Rep, NN	18.2	Medium to large effect reduction in NN for experimental group. 33% remission rate
van Schagen et al [64, 65]	Neth	90 (72 F)	36	Civ Psychiatric	IRT	Passive Active	6 sessions (added to treatment as usual)	Ind	NFQ, NDQ, NES: NN, NNN, DI		Moderate effect reductions in NN, NNN & DI for the IRT group compared to control, held at 3, 6- and 9 m F/U
Walters et al [66]	Aus	55 (0 F)	35	Mil PTSD	PE + IRT + CBT-I	Active PE + SCT	PE = 12 × 90 min IRT = 5 × 60 min CBT-I = 7 × 60 min SCT = 12 × 60 min		CAPS: NN, NNN		Relative to the end of PE (week 6), IRT increased diary-derived with non-sig but medium-large effect size (NM frequency decreased with a large effect size but also did not meet statistical sig)
Woodward et al [67]	UK	121 (71 F)	39	Civ PTSD	CT-PTSD (weekly or daily) Intensive CT-PSTD	Active (Emotional Supp Therapy—EST) + Passive	12weekly over 3 m (standard CT) or daily over 5–7 days (intensive CT)	Ind	NN, NNN		Reductions in NN, NNN greater than supportive therapy & the waitlist, including 40w F/U

*NET* Narrative exposure treatment, *RTM* Reconsolidation of traumatic memories, *CT* Cognitive therapy, *CPT* Cognitive processing therapy, *IR* Image rescripting, *IRT* Image rehearsal therapy, *IRT* + Image rehearsal therapy with sleep hygiene, *BSI* Behavioral sleep intervention, *IE* Imaginal exposure, *PE* Prolonged exposure, *SCT* Supportive care therapy, *ERRT* Exposure, relaxation & rescripting therapy, *DI* Distress, *LTD* Lucid dreaming therapy, *MCC* Minimal contact control, *RTM* Reconsolidation of traumatic memories, *CBT-I* CBT for insomnia, *PCT* Person centered therapy, *NN* Number of nightmares, *NNN* Number of nights with nightmares, *S-Rep* Self-report, *IN* Intensity, *DI* Distress, *DSQ* Daily sleep questionnaire, *NFQ* Nightmare frequency questionnaire, *NES* Nightmare effects survey, *TRNS* The trauma related nightmare survey, *PSS-I* Post-traumatic stress symptom inventory, *NDQ* Nightmare distress questionnaire, *SLEEP-50* Sleep complaints, *NM man* Nightmare management treatment, *Civ* Civilian, *Mil* Military, *Idiopath* Idiopathic nightmares, *pt* Participant, *sig* Significant/ce/ly, *S-Rep* Self-report, *F/U* Follow-up

Of the remaining studies that assessed forms of image rehearsal or rescripting therapies, all but one showed significant reductions in nightmare symptomatology. All of the exposure and exposure plus image rehearsal studies found significant symptom reduction. While the CBT and CBT-I interventions were found to be effective, two of the five studies found exposure or IRT treatments to be superior. The two studies of cognitive processing therapy (CPT) showed significant symptom reduction, while the two studies of lucid dreaming therapy (LDT) showed inferior results to exposure therapy. Psychosocial therapies outperformed passive controls in most studies and were at least equivalent to pharmacological interventions. Group and individual therapies were shown to be equally effective across a range of treatment types, and all but one self-guided therapy study produced significant symptom reduction. Treatment effects were sustained or increased at follow-up in most studies. Effects appeared independent of the sample presentation (idiopathic or part of a more complex presentation).

### Risk of bias (RCTs)

As shown in Table 2, all studies randomly assigned participants to treatment groups, although nearly half of the studies failed to report the randomization technique in sufficient detail for replication. The majority of studies blinded participants, however, several studies reported problems with the blinding process, which may have resulted in participants being aware of their group allocation. All studies were single blinded based on the difficulty of blinding clinicians to the treatment they deliver. There was little evidence of incomplete outcome data and selective reporting, and the majority of studies blinded the outcome assessment. It was noted that many studies failed to report between group effects in the abstract and in concluding, reporting just the within group changes. In a minority of studies, this gave a false impression that reported effects were based on comparisons to the control group. A minority of studies failed to report attrition rates.

**Table 2:** Risk of bias of RCTs examining psychosocial treatments of nightmares

Author (year)	Random Sequence Generation	Allocation Concealment	Blinding (Outcome assessment)	Incomplete outcome data	Selective reporting
Belleville et al [34]	Low	Low	Low	Low	Low
Casement et al [35]	Low	Unclear	Low	Low	Low
Cook et al [36]	Low	Unclear	Low	Low	Low
Davis et al [37]	Low	Unclear	Low	Low	Low
Davis & Wright [38]	Low	Unclear	Low	Low	Low
Forbes et al [39]	Low	Unclear	Low	Low	Low
Germain et al [40]	Low	Unclear	Low	Low	Low
Gieselmann et al [41]	Low	Unclear	Low	Low	Low
Gray et al [42]	Low	Low	Unclear	Unclear	Low
Gutner et al [43]	Low	Unclear	Low	Unclear	Low
Harb et al [44]	Low	Low	Low	Low	Low
Holzinger et al [45]	Low	Unclear	Unclear	Unclear	Low
Krakow et al [33]	Low	Unclear	Low	High	Low
Krakow et al [46]	Low	Unclear	Low	High	Low
Kunze et al [47]	Low	Unclear	Low	High	Low
Lancee et al [48]	Low	Unclear	Low	Low	Low
Lancee et al [49]	Low	Unclear	Low	Low	Low
Lancee et al [50]	Low	Unclear	Low	High	Low
Lancee et al [51]	Low	Unclear	High	Low	Low
Larsen et al [52]	Low	Low	Low	Low	Low
Margolies et al [53]	Low	Unclear	Low	High	Low
Pruiksma et al [54]	Low	Unclear	Low	Low	Low
Pruiksma et al [55]	Low	Unclear	Low	Low	Low
Rhudy et al [56]	Low	Unclear	Unclear	Low	Low
Sheaves et al [57]	Low	Unclear	Low	Low	Low
Spoormaker & van den Bout [58]	Low	Unclear	Low	Low	Low
St-Onge et al [32]	Low	Unclear	Low	Low	Low
Swanson et al [59]	Low	Unclear	Low	Low	Low
Talbot et al [60]	Low	Unclear	Low	Unclear	Low
Taylor et al [61]	Low	Unclear	Low	Low	Low
Thünker & Pietrowsky [62]	Low	Unclear	Unclear	Low	Low
Ulmer et al [63]	Low	Unclear	Low	Low	Low
Van Schagen et al [64, 65]	Low	Unclear	Low	Low	Low
Walters et al [66]	Low	Low	Low	Low	Low
Woodward et al [67]	Low	Low	Low	Low	Low

*Low* Low risk of bias, *high* High risk of bias, *unclear* Unclear risk of bias (not reported or under reported)

### Non-RCTs

The studies reviewed (see Table 3) included 7 case studies, 10 case series, and 16 before and after studies, of which one utilised a control group for part of the study [68]. The case studies were mostly medium (5 – 10 sessions) to long (15+ sessions) in length of treatment, whilst the case series were mostly brief (1- 2 sessions) or short (3 - 4 sessions). The before and after studies included a mixture of different lengths. Effectiveness comparisons by length of treatment did not reveal notable findings. All 34 studies reported an improvement in one or more nightmare symptom, with half of these studies conducting analyses of significance.

**Table 3:** Summary of Non-RCTs examining psychosocial treatments of nightmares

Author	Country	Participants/ Sex/ *N* = female	Mean or Age Range	Population	Study Design	Intervention	Sessions	Mode	Outcome measures	% Attrition	Outcomes
Balliett et al [69]	USA	18 (5 F)	56.6	Mil Vet (no PTSD Dx req)	Uncontrolled Before and After	Modified ERRT	4 × 90–120 min weekly	Ind/Pairs/Both	TRNS: NN, NNN, DI	0	Sig reductions in NN, NNN, & DI from pre to 1w F/U with gains maintained at 2 m F/U. 50% of pts reported cessation of NMs
Berlin et al [70]	USA	1 (0 F)	69	Mil PTSD	Case Study	CBT-I + IRT	60 min monthly 1 × CBT-I 4 × IRT	Ind	NN		Reduction in NN overall. No analyses. No F/U
Bishop et al [71]	USA	14 (1 + F)	49.1	Mil PTSD	Case series	CBT-I + IRT	8 × weekly Tx sessions	Ind	NFQ: NN	21.4	Sig reductions in NN from pre to post and pre to 1 m F/U with large effect sizes
Cavera et al [72]	USA	1 (0 F)	39	Civ PTSD	Case Study	PE	20 × 60 min Weekly	Ind	NN, IN	0	Extinction of NN pre to halfway through Tx & maintained at post, 1 m & 3 m F/U. No analyses
Criswell et al [73]	USA	30 (22 F)	44	Civ PTSD	Uncontrolled Before and After	CBT + IRT for those with NMs	6– 4 sessions weekly (IRT was 1–3 sessions)	Ind	Presence of “distressing dreams”	20	Reduction in percentage of pts experiencing “distressing dreams” from (15/30) 50% pre to (1/26) 4% post and 3/24 (13%) at 3 m F/U. Does not separate pts who received IRT. No analyses of sig
Davis & Wright [74]	USA	4 (3 F)	38.5	Civ PTSD	Case series	Modified IRT + and ERRT	3 × 120 min weekly + 2 × F/U sessions (3 m/6 m)	Ind	TRNS: NN, IN, SE	25	Pre to Post NN extinct for 3 pts & unchanged for the other however reduction in SE (extremely to mildly). At 3 m F/U NN extinct for 2 pts, and unchanged from pre Tx for 2 pts however both with decreases in SE (extremely to mildly). At 6 m F/U, NN extinct for the 3 pts able to be contacted
Davis et al [75]	USA	1 (1 F)	16	Civ PTSD	Case Study	IRT	5 sessions (3 Tx + 2 booster at 1 m/3 m)	Ind	DSAL: NN, IN	0	No changes from pre to post in NN & IN however discovered pt rescripting incorrectly. This was addressed and NN were extinct at 1 m and 3 m F/Us. No analyses
Eakman et al [76]	USA	8 (0 F)	35.6	Mil (75% Posttraumatic stress)	Uncontrolled Before and After	CBT-I + brief IRT	15 × 60 min over 8 weeks	Group/Ind	PSQI-A (does not separate out NM)	14.3	Reportedly sig reduction in “sleep disturbances and nightmares” from pre to post. However, does not measure NM alone. No F/U
Ellis et al [77]	USA	20 (9 F)	43.4	Civ Psychiatric	Case series	Modified IRT at inpatient with pharma/psych support	4 × 60 min over 3 weeks	Group	DDNSI: total, SE, IN	0	NN not stated however sig diff with large effect sizes pre to post for SE, IN, and total scores on DDNSI. No F/U
Fernandez et al [78]	USA	2 (2 F)	8 & 11	Civ PTSD	Case series	ERRT modified for children	4 × Tx sessions	Parent/Child	NDQ/TRNS-C: NN, IN, DI	0	Overall reduction in NN for both pts, one sig Non-sig pt experienced increase in NN throughout Tx until dramatic decrease after re-writing script & psychoed. Non-sig decrease in IN and DI for one pt and unchanged for the other. No F/U
Gellis & Gehrman [79]	USA	11 (0 F)	58.6	Mil PTSD	Uncontrolled Before and After	CBT–I	5 weeks	Ind	NES + NFQ: NNN	27	Non-sig slight reduction in NNN & no change in Nightmare Effects. No F/U
Germain & Nielsen [80]	Canada	12 (5 F)	19 to 58	Civ Mixed (PTSD- NM + idiopath- NM)	Uncontrolled Before and After	IRT	1 × 180 min	Group	NDQ: NN, DI	8.3	At 8.5 weeks post, reductions in retrospective NN for all groups (total, P-NM, I-NM) with medium to large effect sizes but only sig for total. Non-sig increases in prospective NN for all groups (total, P-NM and I-NM) with small to medium effect sizes. Non-sig decreases DI for all groups (total, P-NM and I-NM) with medium to large effect sizes. No F/U
Germain et al [81]	USA	10 (7 F)	33.9	Civ PTSD	Uncontrolled Before and after	IRT +	1 × 90-min	Ind	PSQI-A + PSD	30	Measured number of dreams in general (not NM specifically), their pleasantness and intensity. At 6w post, all 3 categories showed non sig improvements. Non-sig reduction in “night-time PTSD symptoms”. No F/U
Grandi et al [82]	Italy	10 (0 F)	29	NM Dx w/o PTSD	Before and after	Exposure	Self-directed 30-60 min daily for 4w via manual	Ind Self-help	NN, IN	0	Sig reductions in NN & IN compared to being on waitlist, sustained at 4y F/U
Harb et al [83]	USA	11 (0 F)	37.3	Mil PTSD	Uncontrolled Before and After	CBT-I + IRT	T = 7/8 (3 × CBT-I + 4/5 IRT)	Ind	NFQ + sleep diaries: NN, IN	36	At 1 m post, per NFQ (unknown if NN or NNN) slight mean reduction with small effect size. Per sleep diaries, nil changes in NN however decrease in IN and NN of target NM with small effect sizes. No F/U. No analyses of sig
Harb et al [84]	USA	48 (0 F)	59	Mil PTSD	Uncontrolled Before and After	IRT	6 sessions	Group	CAPS: NN, NNN	21	Most effective when the rescripted dream incorporates a resolution of the NM theme and excludes violent details
Hauri et al [85]	USA	36 (17 F)	32.71 (6 – 71) 4 children	DNI Civ/Mil Parasomnias (10 NMs)	Uncontrolled Before and after	Hypnotherapy	1 – 2 × 50 min	Ind	S-Rep on presence or improvement	40 of NM pts	NM pts not commented on alone for 1 m F/U however for all pts able to be hypnotized, 55.5% reported improvement. At 18 m F/U, of the NM pts 5/7 Indicated “Spell Free or Much Improved”. At 5y F/U 4/6 indicated “Spell Free or Much Improved”. No analyses for sig. High attrition
Kovacevic & Davis [86]	USA	1 Trans male	19	Civ PTSD	Case Study	ERRT + CPT w/o PE	5 × ERRT + 12 × CPT	Ind	TRNS/NDQ/NES: NN, SE	0	Reduction in NN & SE from pre to post ERRT with NM extinct by session 7 of CPT. Post CPT weekly non trauma related NM were reported at 3 m & 6 m F/U however SE remained lower than pre. No analyses
Krakow et al [87]	USA	62 (52 F)	40	Civ PTSD NM mixed	Uncontrolled Before and After	IRT +	3 × weekly Tx sessions + 1 F/U (10 h)	Group	NFQ: NN, NNN		Sig reduction in NN & NNN at 3 m F/U
Linden et al [88]	USA	11 (4 F)	12.3	Civ Idiopath(pulmonary patients)	Case series	Self-hypnosis	Unclear	Ind	S-Rep non-directed descriptions of NM/IN		Recurrent NM decreased in frequency or resolved. No statistics. No analyses. No F/U
Long et al [89]	USA	37 (0 F)	62	Mil PTSD	No control	IRET	6 × 90 min	Group	NNN		Large effects for IRET on NNN, & better than control. No F/U
Lu et al [90]	USA	15(0 F)	55	Mil PTSD	No control	IRT	6 × 90 min	Group	NNN, NN, IN	40	No immediate post-treatment effects, sig reduced NNN at 3 & 6 m F/U. High attrition
McNamara et al [91]	USA	19 (10 F)	49.9	Civ (DNS trauma/idiopath)	Uncontrolled Before and After	Virtual reality IRT (ReScript)	2 × weekly for 4 weeks (8 sessions)	Ind	NFQ/NDQ/NES: NNN, DI		Sig reduction in NNN from pre to post (4 weeks) with small effect size. Sig decrease in DI from pre to post with medium – large effect size. No F/U
Miller et al [92]	USA	8 (4 + F, 2 Trans)	36.9	Civ Bipolar Dx with trauma	Uncontrolled Before and After	ERRT-B (addition of bipolar psychoeducation/symptom planning)	5 × 90 min	Ind	TRNS: NN, NNN, SE	14.3	Reductions in mean NN, NNN & SE from pre to post to 3 m F/U with large effect sizes, no comment on significance. NM extinct for 6 of 7 pts at 3 m F/U. SE decreased for all but 1 pt who still reported decrease in NN/NNN
Moore & Krakow [93]	USA	11	-	Mil PTSD	Case series	IRT	4 × 60 min	Ind	NN	0	NN decreased by 44% at 1 m F/U & sig better than control. 4pts showed no improvement. F/U outperformed post-treatment
Nappi et al [94]	USA	58 (9 F)	50	Mil PTSD	Passive	IRT	5 × 60–120 min	Ind/Group	NN, NNN, IN	30	Sig reductions in NN, NNN and IN. 23% complete remission. No F/U
Peirce [95]	USA	1 (0 F)	10	Civ psychotic dx, ASD, ID, trauma but no PTSD Dx	Case study	IRT	5 × IRT sessions over 4 weeks + additional unspecified weekly therapy	Ind	NN via volunteered S-Rep to therapist/teacher		Reduction in NNN from daily to every second day to once a fortnight. No formal stats. No analyses. No F/U
Sheaves et al [96]	UK	7 (4 F)	39.7	Civ idiopath dreams, 3/6 PTSD, all with psychotic symptoms	Case series	IRT w/o exposure	× 4–6	Ind	NN, DI, IN	16.7	Overall slight increase in mean NN and overall reductions in mean DI, IN & vividness. No analyses for sig (lack of power). No F/U
Simbard & Nielsen [68]	Canada	17 (6 + F)	6 to 11	Civ Idiopath	Before & After with partial control. Tx group IRT session 2. Control group received IRT session 3	IRT with drawing	3 × 60–90 min over 8 weeks	Mother–Child/Child	DDLI/NDI/NDQ: NN, IN, DI	35.3	Sig reductions in DI following psycho-ed only (session 1). After session 2, DI decreased further for 4/6 in IRT group & no further for ¾ in control. Following session 2, decreases in NN for 7 pts, stable for 4 pts, and increased for 2pts (groups unknown). Overall, NMs ceased at 3 m F/U for 5/7 pts. NM ceased at 6 m F/U for 3/6 pts
Spoormaker et al [97]	The Netherlands	8 (6 F)	27.8	Civ (DNS trauma/idiopath)	Case series	LTD	1 × 60 min	Ind	NN	0	Reduction in mean NN from pre to 2 m F/U. Only 4 pts were able to become lucid, with 3 able to alter the NM lucidly. NM of 3 other pts changed by itself, i.e. without lucidity”. No analyses for significance
Tufnell [98]	UK	4 (2 F)	4 to 11	Civ PTSD	Case series	EMDR (within multimodal package)	3 -4 EMDR within 5–7 sessions over 2–6 months	Ind/Parent/Both	Presence of NMs	25	NM outcomes only stated for 1 pt (6-year-old male) with no pre NN & DI stated. Per mother – NM extinct at 1 m F/U and maintained at 6 m F/U. No statistics or analyses
Wanner et al [99]	USA	2 (0 F)	58 & 59	Mil PTSD	Case series	ERRT	4 × 60 min weekly	Ind	DSAL: NNN		Reduction in NNN from pre to post for 1 pt with further decline at 3 m F/U. Reduction from pre to 3 m F/U for the other pt but increase from pre to post. Significance not commented on
White [100]	USA	1 (0 F)	20’s	Mil PTSD	Case Study	Embodied Imagination	“several month(s)”	Ind	S-Rep presence of NMs	0	NM reported to become extinct at conclusion. No statistics. No analyses. No F/U
Woo [101]	Singapore	1 (1 F)	36	Civ Idiopath	Case Study	EMDR	4 × 60 min	Ind	NN, DI	0	IN & NN reported to reduce following 1^st^ session. At conclusion dreams still present however without “disturbances”. Doctor report of nil sleep “further” disturbances at 1 m, 3 m, & 5 m F/U

*CPT* Cognitive processing therapy, *IRT* Image rehearsal therapy, *IRT* + Image rehearsal therapy with sleep hygiene, *IRET* Imagery rescripting and exposure therapy, *PE* Prolonged exposure, *ERRT* Exposure, relaxation & rescripting therapy, *EMDR* = Eye movement desensitization and reprocessing treatment, *LTD* Lucid dreaming therapy, *CBT-I* CBT for insomnia, *NM* Nightmares, *NN* Number of nightmares, *NNN* Number of nights with nightmares, *IN* Intensity, *DI* Distress, *SE* Nightmare severity, *DSQ* Daily sleep questionnaire, *PSQI-A* The Pittsburgh Sleep Quality Index Addendum for PTSD, *PSD* The pittsburgh sleep diary, *DDLI* Daily dream log interview, *DSAL* Daily sleep activities log, *DDNSI* The disturbing dreams and nightmares severity index, *NFQ* Nightmare frequency questionnaire, *NES* Nightmare effects survey, *TRNS* The trauma-related nightmare survey, *TRNS-C* The trauma-related nightmare survey – child version, *NDQ* Nightmare distress questionnaire, *NDI* Nightmare distress interview, *Civ* Civilian, *Mil* Military, *Idiopath* Idiopathic nightmares, *pt* participant, *sig* Significant, *S-Rep* Self-report, *F/U* follow-up, *Tx* Treatment, + Actual number of females may be higher as number reported is after attrition

The image rehearsal or rescripting therapies were the most common form of intervention used. Half of these rescripting studies also included CBT-I or select features (e.g., stimulus control, sleep restriction, and/or sleep hygiene) in their treatment delivery. Significant reductions in various nightmare symptomatology were reported in half of the rescripting studies. Six of the remaining studies reported decreases in symptomology however did not conduct analyses of significance. One of these also reported a slight increase in mean frequency of nightmares whilst observing decreases in nightmare distress, intensity, and vividness [69]. The final study found non-significant improvements, however measured dreams rather than nightmares specifically [81]. The only study delivering CBT-I alone provided weak positive results  [79].

Two of the six studies employing mixed exposure and image rehearsal-based therapies through ERRT reported significant improvements in nightmare symptomology. The remaining four studies reported improvements, without measuring statistical significance. Kovacevic and Davis [86] used CPT following ERRT and found nightmare frequency and severity reduced following ERRT and then further decreased until cessation during CPT. Of the two exposure-only studies, the self-directed study found that significant reductions were maintained over 4 years of follow-ups [82] and the other study reported an extinction of nightmares maintained at 1 and 3month follow-ups but did not conduct analyses of significance [72].

The two EMDR studies [98, 101] reported improvements in nightmare symptomology, however, both were third person accounts (mother and doctor), with no formal statistics or analyses. The two hypnotherapy studies reported improvements, however neither used analyses of significance. Furthermore, Hauri et al [85] post assessment did not separate nightmares participants from the other parasomnia participants. The embodied imagination case report [100] declared an extinction of nightmares however did not use statistics or follow up. The lucid dreaming therapy study reported a decrease in mean nightmare frequency, however, the number of participants who were successful in lucidly changing their dreams was the same as the number of participants whose dreams changed without lucidity.

Seven of the 34 studies explored treatments (3 IRT, 1 ERRT, 2 hypnotherapy, and 1 EMDR) in adolescents and children (aged 4 – 16 years). Nightmare symptoms improved in each study, however, Davis et al [75] and Fernandez et al [78] noted the need to rewrite the child’s script before improvements occurred. Interestingly, this coincided with the end of therapy; which meant no further therapist driven exposure work. Also noteworthy was that Hauri et al [85] used a mixed sample of children and adults and did not comment on child outcomes specifically.

Regarding sample presentations, three studies clearly stated the use of civilians with idiopathic nightmares, nine studies included posttraumatic civilian cohorts, and 12 studies included posttraumatic military cohorts. The remaining nine studies consisted of cohorts which were either mixed (idiopathic and posttraumatic), unclear (did not state civilian/military and/or idiopathic/posttraumatic), or complex (e.g., psychiatric) in their presentations. There was a mixture of therapies amongst the different cohorts however, the posttraumatic cohorts (civilian and military) mostly received IRT with CBT-I or its sleep features.

In comparing individual delivery (*n* = 22) with group delivery modes (group = 5, mixed group and individual = 3, and mixed parent-child dyad = 3), the group delivery made up the majority of reported significant improvements in nightmare symptomatology. Many of the individual delivery studies reported improvements but either did not conduct analyses (e.g., all the case reports) or did not report formal statistics.

Finally, many studies collected formal follow up data (*n* = 16), whilst others used informal follow up through third parties (e.g., doctor/parent report; *n* = 2). In most of these studies, treatment gains were sustained or increased. This left 15 studies that did not employ any follow up.

### Risk of bias (non-RCTs)

As shown in Table 4, the majority of studies included participants with the common baseline frequency of nightmares of at least one per week. Those rated as high either used vague terms such as “frequent” or “recurrent” to describe baseline nightmare frequency or included participants with less than 1 nightmare per week, or without nightmares at all. Most studies did not comment on whether participants were receiving concurrent psychological or pharmacological support or only addressed one of these elements. Most studies did not explicitly report on blinding of outcome assessors, or reported that the outcome assessor was also involved in the delivery of the treatment and the in-session assessments. No other blinding was reported. Regarding selective outcome reporting, studies were mixed in their attention to reporting on all outcomes and dropouts. Finally, in considering data sampling in the context of this group of non-RCTs, there was a mixed response. Data collection and reporting methods were considered, with several studies failing to use formal statistics or collection methods. The frequency of data collection (e.g., daily/each session/follow-up), and sample size were considered as part of risk.

**Table 4:** Risk of bias of non-rcts examining psychosocial treatments of nightmares

Author (year)	Participant Selection	Confounds	Blinding	Selective Reporting	Data sampling
Balliett et al [69]	Low	Unclear	Low	Low	Low
Berlin et al [70]	Low	High	Unclear	High	Unclear
Bishop et al [71]	Low	Unclear	Unclear	Low	Low
Cavera et al [72]	Low	Low	Unclear	Low	Unclear
Criswell et al [73]	High	High	High	Unclear	Low
Davis & Wright [74]	Low	Unclear	Unclear	Low	Unclear
Davis et al [75]	Low	Unclear	Unclear	Low	Unclear
Eakman et al [76]	Unclear	Unclear	Unclear	High	Unclear
Ellis et al [77]	Unclear	High	Unclear	High	Low
Fernandez et al [78]	Low	High	High	Low	High
Gellis & Gehrman [79]	Low	High	Unclear	Low	Low
Germain & Nielsen [80]	Low	Unclear	Unclear	High	Low
Germain et al [81]	Unclear	Unclear	Unclear	Unclear	Unclear
Grandi et al [82]	Low	Unclear	Unclear	Low	Low
Harb et al [83]	Low	High	Unclear	Unclear	Low
Harb et al [84]	Low	Low	Low	Low	Low
Hauri et al [85]	Unclear	High	Unclear	High	High
Kovacevic & Davis [86]	Low	Unclear	Low	Low	Unclear
Krakow et al [87]	Low	High	Unclear	Low	Unclear
Linden et al [88]	Unclear	Unclear	Unclear	High	High
Long et al [89]	Low	High	High	Low	Low
Lu et al [90]	Low	High	High	Low	Low
McNamara et al [91]	High	High	Unclear	High	Low
Miller et al [92]	Low	High	Low	Low	Low
Moore & Krakow [93]	Low	High	High	Low	Low
Nappi et al [94]	Low	High	High	Low	Low
Peirce [95]	Low	High	High	Unclear	High
Sheaves et al [96]	Low	Unclear	High	Low	Unclear
Simbard & Nielsen [68]	Low	Low	High	Unclear	Low
Spoormaker et al [97]	Low	Unclear	Unclear	High	High
Tufnell [98]	High	High	High	High	High
Wanner et al [99]	Low	High	High	Low	Unclear
White [100]	High	Unclear	High	High	High
Woo [76]	Low	Unclear	High	High	High

*Low* Low risk of bias, *high* High risk of bias, *unclear* Unclear risk of bias (not reported or under reported)

## Discussion

This review examined the effectiveness of psychosocial treatments for nightmares. In line with evidence hierarchies [102] and the author’s risk of bias assessments, conclusions drawn around efficacy prioritised information gathered from the RCTs over the non-RCTs. Results demonstrated that image rehearsal and rescripting based therapies had the highest quantity of evidence and strong support for their effectiveness in adults (Table 5). Symptom reductions were generally held or improved at follow up. Similarly, there was strong support for the use of exposure-based therapies and ERRT. There was moderate support for the use of CBT based therapies, while the use of LDT, hypnotherapies, and EMDR require more evidence.

**Table 5:** Summary of psychosocial treatment efficacy for nightmares

Treatment	Quantity of evidence	Quality of Evidence	Support for Use
IRT	High	Moderate	High
IR	Low	Moderate	High
RTM	Low	Moderate	High
Exposure	Moderate	Moderate	High
IE	Low	Moderate	High
ERRT	Low	Moderate	High
CPT	Low	Moderate	Moderate
CBT	Moderate	Moderate	Moderate
CBT-I	Moderate	Moderate	Moderate
CT	Low	Moderate	Moderate
LDT	Low	Moderate	Low
HYPNO	Low	Low	Low
EMDR	Low	Low	Low

*IRT* Image rehearsal therapy, *IR* Image rescripting, *RTM* Reconsolidation of traumatic memories, *IE* Imaginal exposure, *ERRT* Exposure, relaxation & rescripting therapy, *CPT* Cognitive Processing therapy, *CBT* Cognitive behavioural therapy, *CBT-I* CBT for Insomnia, *CT* Cognitive therapy, *LTD* lucid dreaming therapy, *HYPNO* Hypnotherapy, *EMDR* Eye Movement desensitization and reprocessing treatment

While most studies reported changes in symptomology with medium to large effect sizes, very few studies reported high remission rates. These findings are consistent with current guidelines where IRT is a recommended treatment along with other “may be used” treatments [19, 20]. It appears that rehearsal and exposure-based therapies specifically target the nightmare content, reducing the emotional arousal associated with the nightmare and/or modifying the content of the nightmare [6]. Harb et al [103] described the core aspects of IRT, as commonly reported in intervention studies, as “choosing a target nightmare, rescripting it, and rehearsing a new dream” (p. 571). This supports Rousseau and Belleville’s [21] review findings where the client’s increased sense of mastery was the mechanism of change in reducing nightmare frequency and intensity. As such, techniques that promote control over the dream content or responses to dream content appear to be most effective for treating nightmares.

There was insufficient evidence to warrant recommendations for promising new (in relation to nightmares) treatments such as CPT and EMDR, however, these treatments would benefit from further high-quality research. Both RCTs assessing CPT [43, 52] reported significant decreases in nightmare symptomology, and the non-RCT study investigating CPT reported an extinction of nightmares [86]. Unfortunately, there were no RCTs of EMDR within the timeframe searched for this review to be able to reevaluate Morgenthaler et al.’s [19] label of EMDR as a treatment to only be “considered”. However, the two non-RCT EMDR studies [98, 101] reported an extinction of nightmares. Hence, RCTs are required to confirm the effectiveness of EMDR.

Findings of the current review suggest that psychosocial treatments have the potential to be beneficial across varied lengths or modes, and for both idiopathic and PTSD nightmares, however further trials and meta-analyses are required. Treatment effects were consistent across gender and age. However, as most studies were US based, research would benefit from investigation of other cultural groups. Treatments as short as two sessions and self-guided treatments produced significant benefits [48, 49, 51]. This suggests that image rehearsal and exposure techniques might be used as part of broader treatment plans for complex presentations such as PTSD. They may be introduced early in treatment to produce relatively quick symptom relief, helping to facilitate subsequent treatment benefits. For this early introduction to be employed with children, and further investigated in RCTs, the appropriateness of the script is important [75, 78]. This includes ensuring that children believe their script, and are not too afraid of the script for its resemblance to the nightmare. After discovering these issues, two non-RCTs reported on the need to rewrite the children’s scripts before observing positive gains [75, 78]. It must also be noted that exposure therapies were found to be effective when delivery was spaced at once or twice a week over 8 weeks, but not effective when delivered once a day for two weeks [61]. Due to the observed flexibility in delivering exposure and image rehearsal techniques in brief, group, and self-guided modes, these modes and techniques may provide clinicians and clients an affordable, efficient, and effective means of reducing nightmare symptoms. More high-quality research on brief and self-guided therapies is needed to support these claims.

One of the difficulties in reviewing studies for the current review was the broad range of often overlapping treatments. For example, IR, IRT, RTM and LDT all involve reimagining the nightmare but differ in other treatment protocols. Similarly, the authors chose to group different but related exposure-based treatment protocols. Furthermore, the non-RCTs demonstrated that protocols within therapies can differ (e.g., IRT or ERRT), with many studies reporting modifications such as removing exposure components [86, 96], lengthening or adding sessions or interventions (i.e., mindfulness) [69], or sometimes drawing instead of writing the dream in the case of children [68]. Similarly, Harb et al’s [103] review of IRT studies found fundamental differences in study quality and protocols. Currently, there is insufficient evidence to explore the nuanced differences in the treatment effectiveness of more specific treatment protocols. Most RCT studies in this review reported that a proportion of participants were using pharmacological treatments for nightmares at baseline, making interaction effects a confounding variable. Due to the high prevalence of nightmares in children, there is an urgent need for RCTs that examine treatment effectiveness in this cohort. The non-RCT child studies demonstrated a variety of data collection methods. It is recommended that future researchers take into account the underestimation effect of parent report and the potential for exaggeration of child self-report by using immediate and frequent reporting. Moreover, it is difficult to determine whether younger children can accurate self-report the difference between distressing dreams and nightmares that awaken the dreamer [104].

It was also noted that some of the studies reviewed made unwarranted conclusions based on within group effects and were potentially misleading. It is therefore recommended that comparison effects between treatments and controls be clearly stated in the abstract. In contrast to a recent review [21], and despite the numerous instruments used to measure nightmare symptoms, measurement of outcomes was not found to be a limitation in evaluating studies. However, there were some non-RCT studies that did not report formal statistics and instead used descriptors such as “recurrent” to “stopped” when explaining nightmare frequency [100]. Overall, most research measured some combination of frequency, intensity, and distress, making comparisons possible. Adverse events were also not recorded or reported in many studies.

There were also limitations associated with the current review. Firstly, due to research team changes, full text inclusion review was conducted by one researcher at a time. As meta-analytic synthesis techniques were not used, combined treatment effects cannot be specified. Additionally, the search strategy was limited to four databases and a specific timeframe which resulted in the most recent research not being included. Risk of bias across studies was not assessed. Finally, we did not specifically synthesize treatment effectiveness for idiopathic and PTSD related nightmares separately. While they did appear to overlap in terms of effective treatments, this may represent limitations in the studies reviewed and in the current synthesis of data. The data tends to homogenize and understate the range of nightmare presentations.

In conclusion, this study systematically reviewed nightmare treatments from 2000-2020. Thirty-five RCTs and 34 non-RCTs were included and provide strong evidence for the efficacy of exposure and image rehearsal-based treatments. These treatments reduced nightmare frequency, severity, and distress, in civilian, military, idiopathic, and PTSD cohorts. There is emerging evidence that self- guided and brief treatment modalities offer efficient and effective treatment options, however more high-quality research is needed. Additionally, there is an urgent need for clinical trials on treatment effectiveness in children. Overall, the results suggest that treatments are most effective when they facilitate a sense of control or mastery by directly targeting the nightmare content and/or the client’s emotional responses to nightmare content.

## Data Availability

On request from corresponding author.
